# Host Specificity for Bacterial, Archaeal and Fungal Communities Determined for High- and Low-Microbial Abundance Sponge Species in Two Genera

**DOI:** 10.3389/fmicb.2017.02560

**Published:** 2017-12-20

**Authors:** Maryam Chaib De Mares, Detmer Sipkema, Sixing Huang, Boyke Bunk, Jörg Overmann, Jan Dirk van Elsas

**Affiliations:** ^1^Microbial Ecology Cluster, Groningen Institute for Evolutionary Life Sciences, University of Groningen, Groningen, Netherlands; ^2^Laboratory of Microbiology, Wageningen University, Wageningen, Netherlands; ^3^Leibniz-Institut Deutsche Sammlung von Mikroorganismen und Zellkulturen, Braunschweig, Germany; ^4^German Centre of Infection Research (DZIF), Partner site Hannover-Braunschweig, Braunschweig, Germany

**Keywords:** *Aplysina*, *Dysidea*, microbial diversity, three-domain microbial communities, sponges

## Abstract

Sponges are engaged in intimate symbioses with a diversity of microorganisms from all three domains of life, namely Bacteria, Archaea and Eukarya. Sponges have been well studied and categorized for their bacterial communities, some displaying a high microbial abundance (HMA), while others show low microbial abundance (LMA). However, the associated Archaea and Eukarya have remained relatively understudied. We assessed the bacterial, archaeal and eukaryotic diversities in the LMA sponge species *Dysidea avara* and *Dysidea etheria* by deep amplicon sequencing, and compared the results to those in the HMA sponges *Aplysina aerophoba* and *Aplysina cauliformis*. *D. avara* and *A. aerophoba* are sympatric in the Mediterranean Sea, while *D. etheria* and *A. cauliformis* are sympatric in the Caribbean Sea. The bacterial communities followed a host-specific pattern, with host species identity explaining most of the variation among samples. We identified OTUs shared by the *Aplysina* species that support a more ancient association of these microbes, before the split of the two species studied here. These shared OTUs are suitable targets for future studies of the microbial traits that mediate interactions with their hosts. Even though the archaeal communities were not as rich as the bacterial ones, we found a remarkable diversification and specificity of OTUs of the family Cenarchaeaceae and the genus *Nitrosopumilus* in all four sponge species studied. Similarly, the differences in fungal communities were driven by sponge identity. The structures of the communities of small eukaryotes such as dinophytes and ciliophores (alveolates), and stramenopiles, could not be explained by either sponge host, sponge genus or geographic location. Our analyses suggest that the host specificity that was previously described for sponge bacterial communities also extends to the archaeal and fungal communities, but not to other microbial eukaryotes.

## Introduction

Sponges (phylum Porifera) feature an intimate symbiosis with diverse microorganisms (Taylor et al., 2007; Simister et al., 2012). Although some species occur in freshwater, we here place a focus on marine sponges. These are sessile filter feeders that acquire organic matter and microorganisms from seawater by pumping large volumes of water through their aquiferous system (Vogel, 1977). In the process, the acquired microorganisms become transferred into the mesohyl tissue, where they are eventually ingested by archaeocytes (Simpson, 1984). Microorganisms can, however, also survive in the mesohyl tissue and become established as part of the sponge-associated microbiota. This process of translocation of microorganisms from seawater to the sponge is referred to as horizontal transmission. Alternatively, sponges may acquire their associated microbiota by means of vertical transmission, through incorporation of microbial cells in egg cells or other reproductive stages. Vertical transmission in sponges has been observed for Bacteria (Ereskovsky et al., 2005; Enticknap et al., 2006; De Caralt et al., 2007; Lee et al., 2009), Archaea (Sharp et al., 2007; Steger et al., 2008) and even yeasts (Maldonado et al., 2005). Consequently, the acquisition of similar sponge-associated microbes can occur both vertically and horizontally (Sipkema et al., 2015).

Microorganisms enclosed within the mesohyl are physically separated from the surrounding seawater by the sponge pinacoderm. Sponges have been documented to harbor a complex community comprising microbial representatives from all three domains of life, the Bacteria, Archaea, and Eukarya, and can contain viruses as well. To date, thousands of bacterial and archaeal species have been identified within this association, being spread over more than 52 phyla (Schmitt et al., 2011; Webster and Taylor, 2012; Thomas et al., 2016). In addition, several microbial eukaryotes including Fungi (Lee et al., 2001; Usher et al., 2007; Usher, 2008; Webster and Blackall, 2009; Simister et al., 2012; Rodríguez-Marconi et al., 2015) have been reported.

Sponges differ in the numbers of bacteria they harbor. Sponges with dense tissues, and poorly irrigated ones, contain larger numbers of bacteria than well-irrigated ones. These different sponge types are referred to as high microbial abundance (HMA) or “bacteriosponges,” and low microbial abundance (LMA) sponges, respectively (Hentschel et al., 2006). HMA sponges have densities between 10^8^ and 10^10^ of bacterial cells per gram of wet sponge weight, resulting in many cases in up to 40% of the sponge biomass being composed of microorganisms (Vacelet and Donadey, 1977; Usher et al., 2004), while LMA sponges have densities around 10^5^ bacterial cells per gram of wet sponge weight, comparable to those of seawater (Hentschel et al., 2006; Gloeckner et al., 2014). Certain bacterial clades are only found in sponges. These “sponge-specific clusters” are defined as groups containing at least three sequences that: (i) are recovered from different sponge species and/or different geographic locations, (ii) are more closely related to each other than to any other sequence from non-sponge sources, and (iii) cluster together irrespective of the method used (Hentschel et al., 2002). Sponge-specific clusters of bacteria should be regarded as integral parts of the sponge, and these are generally harbored by HMA sponges, whilst LMA sponge species are thought to have less specifically associated microbiota (Webster et al., 2004; Giles et al., 2013), as well as a lower diversity of associated microorganisms. However, deeper sequencing has recently revealed that bacteria previously thought to be confined to sponges may also be present in seawater at low abundances (Taylor et al., 2013). This could be the product of the liberation of bacteria upon the death of sponge cells, or could be caused by a broader distribution of bacteria that survive in the water, but are enriched specifically within the sponge hosts.

Sponge-associated archaeal communities appear to follow the HMA-LMA dichotomy found in Bacteria, with typically up to four orders of magnitude higher numbers in HMA over LMA sponges (Bayer et al., 2014). In sponges, the domain Archaea is represented by few specific clades, mainly related to *Cenarcharchaeum symbiosum* (phylum Thaumarchaeota). This phylum dominates sponge-associated microbial communities in sponges from the Arctic (Pape et al., 2006) and Irish deep-sea environments (Jackson et al., 2013). In addition, LMA sponges in some cases harbor as many Archaea as HMA sponges, as is the case for *Stylissa carteri* from the Red Sea (Bayer et al., 2014).

Marine eukaryotic microorganisms are both diverse and important components of ocean ecosystems; however, they are understudied compared to other microorganisms. In sponges, most of the fungal diversity has been found to comprise yeast-like organisms. In contrast to the bacterial communities associated with sponges, the existence of sponge-specific fungi is debatable (Rodríguez-Marconi et al., 2015; Naim et al., 2017). Other microbial eukaryotes such as the “SAR” supergroup—a clade that includes stramenopiles, alveolates and Rhizaria (Burki et al., 2007)—have been encountered in sponges (Webster et al., 2004; Sipkema and Blanch, 2009). However, such micro-eukaryotic communities do not seem to associate specifically with sponges (Rodríguez-Marconi et al., 2015).

The total microbial diversity, including Bacteria, Archaea and microbial eukaryotes of the same sponge species has very rarely been studied. Moreover, little is understood about the specificity of the microbial associations within a single sponge genus across different geographic locations. In this study, we investigated the microbial diversity of the LMA sponge species *Dysidea avara* and *D. etheria* and compared it with that of the HMA sponge species *Aplysina aerophoba and A. cauliformis*. We hypothesized that the diversity and community structure of bacterial and archaeal microbial communities of the HMA genus *Aplysina* would be better preserved across geographic locations than those of the LMA genus *Dysidea* and that the eukaryotic communities would not be preserved across geographic locations in either genus. To investigate host specificity and to identify intra- and inter-species variation, we sampled individuals of all sponge species in triplicate, as well as seawater from both geographic locations.

## Materials and methods

In the Mediterranean Sea, sponge samples were collected by SCUBA diving in April 2014 in Cala Montgó, Spain (42°06′ 52.6″ N, 3°10′ 02.0″ E). Specimens of *A. aerophoba* were collected from 7.8 to 12.7 m depth, and specimens of *D. avara* at 7.2 m depth. Individual samples were collected in 50 mL sterile tubes after *in situ* identification (Supplementary Table 1). Habitat seawater was also collected at the same location in a sterile container to compare sponge-associated bacteria to bacterioplankton. Samples were transported on ice, and approximately 2 h after sampling upon arrival to the laboratory at Centre d'Estudis Avançats de Blanes (Spain), sponges were washed three times with sterile artificial seawater. Then, 1 cm^3^ pieces were cut from each individual and stored in RNALater (Ambion) until DNA isolation. At the Florida Keys in the Caribbean, the same sampling methods were applied in May 2014. *D. etheria* individuals were collected in Summerland Key (24° 39′ 36″ N, 81° 27′ 36″ W), and *A. cauliformis* samples were collected in Long Key (24° 49′ 48″ N, 80° 46′ 12″ W).

### DNA extractions, PCR amplifications and sequencing

Total genomic DNA was extracted in triplicate from sponge tissues (150–200 mg) and seawater using the FastDNA® Spin Kit for Soil (Q-Biogene, Carlsbad, CA) according to the protocol of the manufacturer. The sponge tissue was sampled including tissue from both endodermal and ectodermal tissues. For the isolation of DNA from planktonic cells, 3 L of seawater were filtered onto a 0.22 μm polycarbonate filter (Millipore) and total genomic DNA was extracted from the filter. In the Caribbean Sea, 500 mL of seawater were filtered. The concentration of extracted DNA was determined with a Nanodrop 1000 spectrophotometer (Nanodrop Technologies, Wilmington, DE), whereas its integrity was visually examined by gel electrophoresis on a 1% (w/v) agarose gel stained with ethidium bromide. The extracted DNA was dissolved in TE buffer and stored at −20°C until further analysis.

Amplicon sequencing of phylogenetic marker genes was conducted for all three individuals of *A. aerophoba, A. cauliformis, D. avara*, and *D. etheria*, as well as for seawater. We used Bacteria and Archaea-specific 16S rRNA gene primers published previously (Klindworth et al., 2013). Archaea—specific primers amplified a 492 bp DNA sequence containing the V5-V6 regions of the rRNA gene using primers Arch934F: 5′-AGGAATTGGCGGGGGAGCA-3′ and UA1406R: 5′-ACGGGCGGTGTGTRCAA-3′. For Bacteria, the V3-V4 region of the 16S rRNA gene was targeted and a 428 bp PCR product was obtained with primers 341F: 5′-CCTAYGGGRBGCASCAG-3′ and 806R: 5′-GGACTACNNGGGTATCTAAT-3′. For characterizing the fungal communities, we amplified the ITS2 region using primers ITS3 (5′-GCATCGATGAAGAACGCAGC-3′) and ITS4 (5′-TCCTCCGCTTATTGATATGC-3′) (Liu et al., 2012), suggested as the standard for fungal community analyses (Bates et al., 2013). The ITS region, however, is known to evolve rapidly and be variable in size (Bates et al., 2013), therefore we selected fragments below 500 bp by excision from gel. Finally, we used a 500-bp 18S rRNA gene fragment to characterize unicellular Eukaryota, including, but not restricted to, Fungi. The primers used, Euk1Af (5-CTGGTTGATCCTGCCAG-3) and Euk516r (5-ACCAGACTTGCCCTCC-3) were selected based on previous studies of marine unicellular eukaryotes (Dìez et al., 2001; Wilms et al., 2006).

PCR amplifications were performed in triplicate for each DNA extraction in a volume of 25 μl containing 1 × MyTaq^TM^BIOLINE buffer, 2.5 mM magnesium chloride, 0.25 mM dNTP mixture, 0.5 U MyTaq^TM^BIOLINE DNA polymerase (BIOLINE), 0.5 μM of each primer and 20 ng template DNA. PCR was performed using an initial denaturation at 94°C for 2 min, followed by 30 cycles of denaturation at 94°C for 30s, annealing at 55°C for 30s (except Bacteria at 45°C), elongation at 72°C for 1 min, and a final elongation at 72°C for 5 min. Amplification products were checked on a 1% (w/v) agarose gel. PCR-products were purified using the Wizard® SV Gel and PCR Clean-Up System (Promega, Wisconsin, USA). Subsequently, PCR products were quantified using the Quant- iTdsDNA high-sensitivity assay kit (Invitrogen, Grand Island, NY).

The PCR products from each sample and each separate microbial community were barcoded prior to sequencing. Libraries for amplicon sequencing were prepared using the NEB Ultra DNA Library Prep Kit for Illumina (NEB, Ipswich, MA, USA) and sequencing was performed on the MiSeq Desktop Sequencer (llumina Inc., San Diego, CA, USA) for 300 cycles in both directions. In total, six MiSeq lanes were sequenced. Bacterial amplifications from Caribbean seawater samples were unsuccessful and therefore removed from further analyses. Sequence data have been deposited in the European Nucleotide Archive under accession number PRJEB22033.

### Sequence processing, taxonomic assignment and diversity analyses

To perform a hierarchical taxonomic rank assignment for each sequence, a pipeline developed by the Leibniz-Institut DSMZ (Esposito et al., 2015) was implemented to pre-process raw sequences. Only sequences that contained intact barcode and primer sequences were included in the analyses. Additionally, sequences containing ambiguous bases were removed from the analyses. A quality filter with a Phred score of 20 was applied. After trimming, quality-filtering and pairing the raw reads, 29,064,287 sequences for Bacteria and Archaea and 8,009,549 sequences were obtained for eukaryotes including Fungi. Bacterial and Archaeal sequences were checked for potential chimeras using UCHIME (Edgar et al., 2011). A taxonomic-independent analysis was conducted for all four communities. Sequences that passed the pre-processing steps were first binned into operational taxonomic units (OTUs) using the open-reference pipeline for OTU picking (Caporaso et al., 2010b) at 97% similarity using UCLUST (Edgar, 2010), followed by picking a representative sequence for each OTU. Representative sequences were aligned to the Greengenes Core reference alignment (DeSantis et al., 2006) using PyNAST (Caporaso et al., 2010a), and the resulting sequences were kept for building the OTU tables. Then, OTUs were taxonomically assigned using the Ribosomal Data Project (RDP) Classifier (Cole et al., 2009) command line version 2.10.1. Sequences were considered as classified at the deepest taxonomic rank with a minimum confidence value of 0.5. Taxonomic assignment of the reads showed that bacterial primers also amplified archaeal phyla. These archaeal reads were discarded for the bacterial OTU analyses and all the bacterial reads for the archaeal OTU analyses.

High-quality fungal ITS sequencing data were processed using the same pipeline. Chimeric sequences were removed using a combination of reference-based and de novo chimera detection with USEARCH 8 (Edgar, 2010); only reads detected as chimeric with both approaches were discarded. For the subsequent analyses, the ITS2 region was extracted from each sequence using Fungal ITS extractor (Nilsson et al., 2010) (http://microbiology.se/software/itsx/), as the conserved flanking regions are known to distort similarity searches, taxonomic assignments and clustering results (Bruns and Shefferson, 2004). The resulting ITS2 reads were clustered to operational taxonomic units (OTUs) by UCLUST (Edgar, 2010), with a 100% similarity threshold to reference sequences. As the reference database, we used the UNITE+INSDC data sets, accessed 22 February 2015 (Abarenkov et al., 2010). Singletons (clusters containing only one read) were discarded as they are indistinguishable from sequencing errors (Kunin et al., 2010). OTUs were identified taxonomically by a local BLAST search against the UNITE+INSDC data set using blast-2.2.30 (http://blast.ncbi.nlm.nih.gov/Blast.cgi) with default settings.

High-quality eukaryotic sequences were taxonomically assigned using SILVA (Pruesse et al., 2007), aligned against the SILVA database release 108 (http://www.arb-silva.de/documentation/background/release-108). Non-aligned sequences were removed from the analysis. Subsequently, remaining sequences were dereplicated, clustered and classified. Dereplication, where identical reads are ignored, was performed using custom Python scripts (available at https://github.com/mchaib/Eukaryote-community.git) with an identity criterion of 1.00. Taxonomic classification was performed by a global search against the SILVA SSURef 108 NR dataset (http://www.arb-silva.de/projects/ssu-ref-nr/) using USEARCH 8 (Edgar, 2010) with default settings.

### Statistical analyses

We conducted a taxonomic-independent analysis based on OTUs for all four microbial communities. To normalize the data for each sample, OTU counts were divided by the total counts of all OTUs within that sample and multiplied by 1000, resulting in relative abundance (RA) expressed as per mille. A log_2_ transformation (log_2_(RA + 1)) of the data was performed for statistical comparisons. We used an inclusion threshold >0.1% relative abundance for an OTU in at least one sample. Normalization, transformation and thresholding were performed using R scripts modified from Bulgarelli et al. (2012). To identify differentially abundant OTUs in the microbial communities between the tested habitats we used linear statistics on relative abundance values (log_2_-transformed, >1% threshold, rarefied) within the R package “limma”. For each OTU, we fitted a linear model with the habitat as explanatory variable. Differentially abundant OTUs between two habitats were calculated using moderated *t*-tests, an approach suited when the number of measurements per sample is large but sample sizes are small. The resulting *p*-values were adjusted for multiple hypothesis testing using the Benjamini-Hochberg false-discovery-rate (FDR) correction method, where significant enrichment was determined at *p*-values ≤ 0.05. To detect OTUs that were more abundant (enriched) in one habitat (sponge or seawater) compared to the other habitats, the intersection of corresponding pairwise comparisons was taken (as described by Bulgarelli et al., 2012).

Log_2_ transformed relative abundance values were used to calculate a Bray-Curtis dissimilarity matrix using the function “vegdist” of “Vegan.” This matrix was used to generate dendrograms using “hclust” within “Vegan”, specifying the average clustering method. Variation in composition among sites was assessed using non-metric multidimensional scaling (NMDS) based on the Bray-Curtis dissimilarity matrix. The NMDS plots were generated using the metaMDS() function from the vegan package v 2.4-2. Prior to the PCO, the raw data were log (x+1) transformed and used to produce a distance matrix with the index with the vegdist() function in vegan. The procrustes() function in vegan was used to assess congruence among bacterial and microeukaryotic PCO ordinations. Default values were used for the arguments in the procrustes() analysis. In addition to the procrustes() function, the protest() function in vegan was used to estimate the significance of the Procrustes statistic. The number of permutations in the protest() function was set to 999. The R vegan adonis() function for permutational multivariate analysis of variance (PERMANOVA) was used to test for significant variations in composition between sample groups. In the analysis, the Bray-Curtis distance matrix of OTU composition was the response variable with samples as independent variables. The statistical significance of the *F*-test was assessed with 999 permutations.

### Identification of sponge-specific and sponge/coral-specific clusters

Representative sequences of OTUs identified as enriched in a particular sponge host were taxonomically assigned using a BLAST (Altschul et al., 1997) search against the curated ARB-SILVA database containing 178 previously-identified sponge-specific clusters (SC) and 32 sponge/coral-specific clusters (SCC), kindly provided by the authors of that study (Simister et al., 2012). An OTU was only assigned to a cluster if it was more similar to the members of that cluster than to sequences outside the cluster and its similarity to the most similar sequence within that cluster was above 75% (as described in Thomas et al., 2016).

## Results

### Variation in bacterial community composition

**Figure 1:**
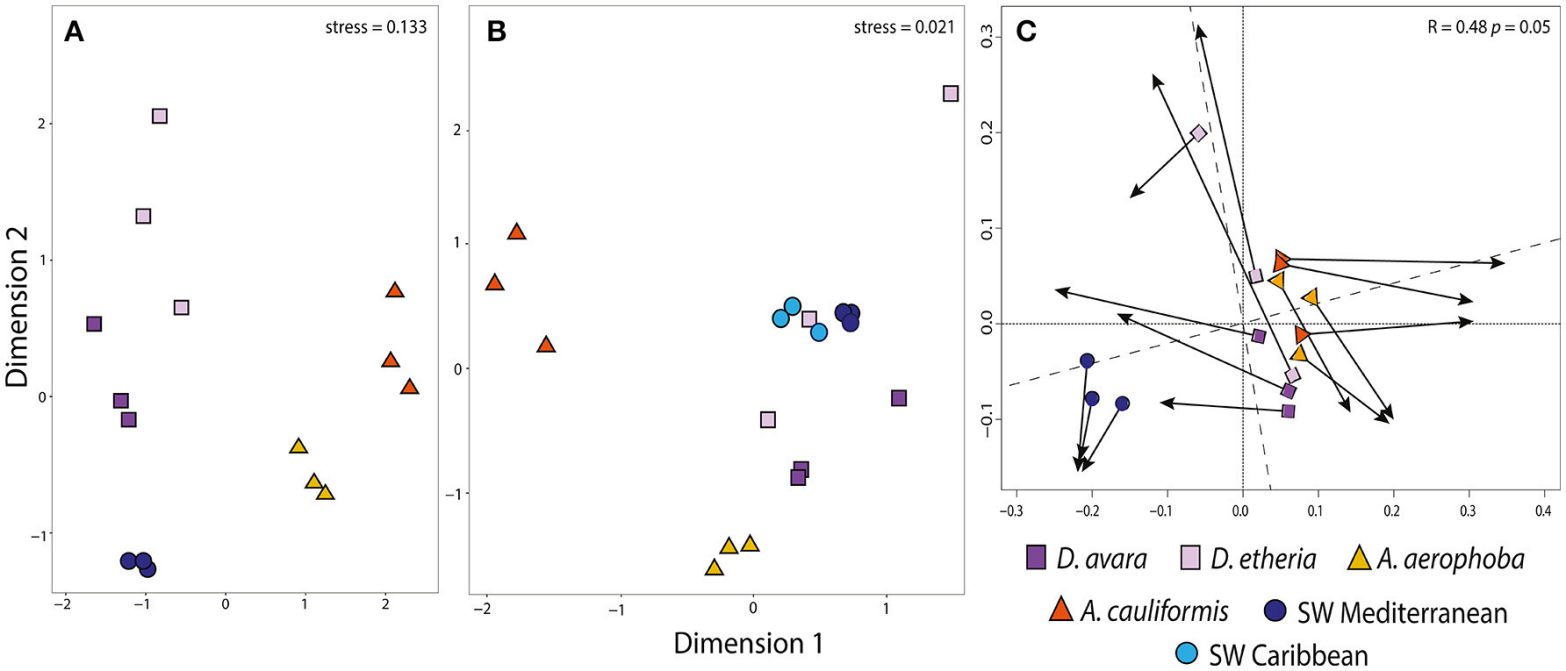
Community structure in prokaryotic communities among all sampled habitats. Ordination showing the first two axes of the NMDS analysis for **(A)** bacterial OTU composition; **(B)** archaeal OTU composition and **(C)** Procrustes analysis comparing bacterial **(A)** and archaeal **(B)** OTU composition. In Procrustes analysis, the arrows point to the target configuration (bacterial OTU composition), the symbols represent the rotated configuration (archaeal composition). Correlation (Corr: 0.48) and significance values (P 0.05) were calculated using the protest function from the vegan R package. Sample groups correspond to *Aplysina aerophoba, A. cauliformis, Dysidea avara, D. etheria* and Mediterranean and Caribbean seawater. Colors for each sample group are specified in the color legend.

To compare the bacterial and archaeal community structures, we calculated Bray–Curtis distances at OTU level and generated NMDS ordination plots (Figures 1A,B). For associated Bacteria, the analysis clearly showed an effect of the host sponge species, with all samples from a given sponge species clustering together. *Aplysina* samples from both geographic locations were clearly separated along the first (horizontal) axis of variation from the rest of the samples (Figure 1A). Along the second axis of variation, there was a strong separation between (Mediterranean) seawater samples and all sponge samples, as well as separations between the different sponge species. adonis analysis revealed a significant difference in bacterial composition between sponge species (adonis: *F* = 3.334, *R*^2^ = 0.571, *P* = 0.01), genus (adonis: *F* = 1.427, *R*^2^ = 0.364, *P* = 0.01), and geographic location (adonis: *F* = 2.521, *R*^2^ = 0.109, *P* = 0.05). Similarly, hierarchical clustering analyses showed bacteria from each sponge species and genus to cluster separately (Supplementary Figure 1A).

The highest bacterial phylum-level diversity was observed in *A. aerophoba*, with 36 different bacterial phyla, followed by Mediterranean seawater with 31 bacterial phyla, and *A. cauliformis* with 27 different phyla. *D. avara* and *D. etheria* had the lowest diversity with 22 and 25 phyla, respectively. At the OTU level, sequences were rarefied to a minimum depth of 8,450 reads per sample (lowest number of reads in a sample) after clustering into 5,044 OTUs (Table 1, Supplementary Tables 2, 3). Chao1 values show that α-diversity values in both *Aplysina* host species and the seawater were significantly higher (*F* = 20.35, *P* = 8.55e−05) than those observed for *Dysidea* host species, confirming previous findings stating that LMA sponge species have lower microbial abundance and also lower richness, as compared to HMA sponge species. Overall, our results show that a large number of bacterial OTUs cannot be classified confidently at deeper taxonomic categories. In fact, from 5,044 OTUs, many could not be classified further than Class level, with 32.8% of OTUs not classified at order (1,654), 51.8% OTUs not classified to family (2,615), and up to 87.2% not classified to genus (4,400) level.

**Table 1:** Number of filtered reads for each community and observed OTUs in seawater and sponge samples at a 97% sequence similarity threshold for prokaryotes and microeukaryotes and 98.5% sequence similarity threshold for fungi (ITS2).

**Sample name**	**Abbrev**.	**Sponge genus**	**No. of bacterial reads**	**No. of archaeal reads**	**No. of fungal reads (ITS2)**	**Total no. of microeukarya reads**	**No. of microeukarya reads not Porifera**	**Bacterial reads**		**Archaeal reads**		**Fungal reads**		**Microeukarya reads**	
								**Observed OTUs**	**Expected OTUs (Chao1)^a^**	**Observed OTUs**	**Expected OTUs (Chao1)^a^**	**Observed OTUs**	**Expected OTUs (Chao1)^a^**	**Observed OTUs**	**Expected OTUs (Chao1)^a^**
**Mediterranean Sea**															
*A. aerophoba* 1	Aa1	*Aplysina*	34,209	1,789,477	279,210	395,294	2,173	1,197	12, 425 ± 227	213	264 ± 23	135	242 ± 40	70	100 ± 16
*A. aerophob*a 2	Aa2	*Aplysina*	41,947	1,226,092	352,172	402,403	3,106	1,302	12, 621 ± 160	160	207 ± 30	167	237 ± 24	53	91 ± 22
*A. aerophoba* 3	Aa3	*Aplysina*	37,018	2,633,717	334,216	447,478	7,626	1,226	12, 939 ± 225	219	248 ± 12	112	158 ± 20	70	101 ± 18
*D. avara* 1	Da1	*Dysidea*	46,412	2,017,172	206,914	447,676	5,376	681	3, 397 ± 69	349	373 ± 11	193	292 ± 31	91	107 ± 9
*D. avara* 2	Da2	*Dysidea*	31,315	2,068,560	181,295	436,855	88,166	876	4, 386 ± 114	235	263 ± 11	244	358 ± 32	100	118 ± 10
*D. avara* 3	Da3	*Dysidea*	28,120	2,010,458	142,302	464,741	9,693	1,208	4, 032 ± 63	192	205 ± 7	27	49 ± 17	107	130 ± 13
*Seawater Mediterranean* 1	SWM1		116,282	2,625,898	224,261	535,350	499,579	719	14, 548 ± 225	394	401 ± 6	328	515 ± 48	159	159 ± 0
*Seawater Mediterranean* 2	SWM2		108,192	2,845,300	308,227	469,621	466,658	707	14, 029 ± 235	390	399 ± 7	393	575 ± 41	158	168 ± 10
*Seawater Mediterranean* 3	SWM3		130,946	3,097,608	200,975	471,441	465,064	745	14, 543 ± 192	399	407 ± 6	338	491 ± 36	159	159 ± 1
**Caribbean Sea**															
*A. cauliformis* 1	Ac1	*Aplysina*	318,385	14,984	567,278	1,196,853	83,779	1,147	14, 942 ± 232	238	255 ± 6	103	138 ± 15	25	31 ± 7
*A. cauliformis* 2	Ac2	*Aplysina*	385,282	18,047	507,567	835,488	417,744	1,226	15, 639 ± 231	367	374 ± 3	87	178 ± 42	27	27 ± 0
*A. cauliformis* 3	Ac3	*Aplysina*	1,493,750	249,426	9,971	149,481	8,969	781	17, 258 ± 109	370	452 ± 23	72	126 ± 28	86	104 ± 13
*D. etheria* 1	De1	*Dysidea*	8,450	84,252	160,818	155,462	137,611	837	1, 980 ± 90	292	309 ± 9	77	146 ± 29	49	55 ± 5
*D. etheria* 2	De2	*Dysidea*	93,116	5,677	229,666	123,956	44,040	832	4, 031 ± 18	268	3 ± 2	77	114 ± 16	65	73 ± 6
*D. etheria* 3	De3	*Dysidea*	15,176	45,848	192,127	131,040	13,050	866	2, 056 ± 87	2	305 ± 17	100	223 ± 48	62	73 ± 6
*Seawater Caribbean* 1	SWC1		0	2,300,482	196	350,760	334,212			542	598 ± 19	16	26 ± 11	66	77 ± 10
*Seawater Caribbean* 2	SWC2		0	1,851,259	101,978	252,887	252,868			443	501 ± 19	222	378 ± 43	30	31 ± 1
*Seawater Caribbean* 3	SWC3		0	2,294,583	15,629	284,003	274,362			563	632 ± 22	62	147 ± 44	70	73 ± 3

Even though host sponge species and genus diverged in taxonomic composition, as seen in the top 21 most (relatively) abundant OTUs (Supplementary Figure 2), they also shared several OTUs. The two *Aplysina* species shared 44 OTUs, assigned at the phylum level to Acidobacteria (19), Proteobacteria (11), Chloroflexi (9), Gemmatimonadetes (3), Bacteroidetes (1), and Deinococcus-Thermus (1), while only two different OTUs were shared between the two *Dysidea* host sponge species. The deepest taxonomic assignment possible for these two shared OTUs was the class Alphaproteobacteria. All OTUs shared by the sponge genera fell into monophyletic clusters of “sponge-specific” (SC) or “sponge- and coral-specific” (SCC) 16S rRNA gene sequences, as defined previously by Simister et al. (2012) (Supplementary Table 6). The two OTUs shared by the two *Dysidea* species matched SCC26 within the Alphaproteobacteria using the SC 16S rRNA gene sequence database. However, an inspection using BLASTn to the nr database revealed that these two OTUs are 98% identical to uncultured Proteobacteria present in seawater, as shown by their closest matches HM474893.1 and JX405457.1 (Supplementary Table 6).

We defined sets of OTUs that correspond to symbionts that were potentially acquired prior to the separation of the lineages leading to the sponge species for each genus studied here, based on the intersection of pair-wise comparisons of OTUs enriched in the different sponges and those in the seawater. At the sponge species level, *A. aerophoba* samples had the largest number of enriched OTUs (438). These belonged to the phyla Chloroflexi (269), Proteobacteria (93), Acidobacteria (28), Actinobacteria (16), Gemmatimonadetes (8), Nitrospirae (7), Spirochaetes (3), Deinococcus-Thermus (1), Bacteroidetes (1) and candidate phyla AncK6 (3), PAUC34f (3), Poribacteria (3) and SBR1093 (2) (fdr < 0.05; Supplementary Table 4). Similarly, we identified 111 OTUs enriched in *A. cauliformis* within the Acidobacteria (26), Actinobacteria (26), Proteobacteria (22), Chloroflexi (15), Bacteroidetes (10), Gemmatimonadetes (9), Deinococcus-Thermus (1), Spirochaetes (1), and the candidate phylum TM7 (1). Among the LMA sponges, *D. avara* harbored 188 enriched OTUs that belong to the phylum Proteobacteria, in particular the Alphaproteobacteria orders Rhodobacterales, Rickettsiales and Kilionellales, and 1 OTU assigned to Bacteroidetes. Likewise, significantly enriched OTUs (33) in *D. etheria* belonged mainly to the phylum Proteobacteria and three were Bacteroidetes (fdr < 0.05; Supplementary Table 5).

### Variation in archaeal community composition

Using the Bacteria-specific primers, only 6% of the reads (192,917 reads) were classified as Archaea, while when using the archaea-specific primers this proportion was 89% (25,666,299) of the high-quality reads. After removal of the bacterial sequences from the archaea-specific primer dataset, the remaining sequences were binned into 65,435 OTUs belonging to 8 different archaeal phyla. The phyla Crenarchaeota, Euryarchaeota, Thaumarchaeota and Woesearchaeota were present in all samples, while Aenigmarchaeota, Aigarchaeota, Diapherotrites and Pacearchaeota were consistently found in low numbers only in the replicates of the Mediterranean seawater samples. After applying the inclusion threshold >0.1% relative abundance for an OTU in at least one sample, only 967 OTUs were kept for subsequent analyses (Supplementary Tables 7, 8). Of these, 6 were not classified at the order level, 47 were not classified at family level, and 685 were not classified at the genus level (Supplementary Table 8). At the OTU level, sequences were rarefied to a minimum depth of 12,694 reads per sample. Based on Bray–Curtis distances at the OTU level, archaeal communities from HMA sponge species clustered together for each of the *Aplysina* species and were distinct from the control seawater, while the LMA samples were more dispersed (Figure 1B; Supplementary Figure 1B). HMA samples were separated from the rest along the first axis of variation. The second axis shows the separation of *A. aerophoba* samples from the rest of the samples. Seawater samples occupy an intermediate position, although the samples from both geographic locations are separated from each other. *Dysidea* samples from both species were the most variable within groups. Based on adonis analyses, significant differences in composition were confirmed for sponge species (adonis: *F* = 7.803, *R*^2^ = 0.438, *P* = 0.01), genera (adonis: *F* = 8.916, *R*^2^ = 0.335, *P* = 0.01) and geographic location (adonis: *F* = 5.202, *R*^2^ = 0.098, *P* = 0.01).

The family Cenarchaeaceae was overwhelmingly dominant in the sponge species studied here. However, within this taxon different OTUs were significantly enriched for each host sponge species. For example, in *A. aerophoba*, 41 enriched OTUs were assigned to the family Cenarchaeaceae and 39 of these were further assigned to the genus *Nitrosopumilus*. In *A. cauliformis*, the majority of OTUs were assigned to Cenarchaeaceae (21), and only 10 of these were *Nitrosopumilus* (Supplementary Table 9). In *D. avara*, there were 8 Cenarchaeaceae OTUs, and in *D. etheria* two OTUs affiliated to the Phylum Euryarchaeota, class Thermoplasmata (Marine group II) (Supplementary Table 10; Supplementary Figure 3). None of these enriched OTUs, even though they belong to the same family (Cenarchaceae) and some to the same genus (*Nitrosopumilus*), were shared between all sponge species included in this study. In order to determine if there was any congruence between the trends observed from the bacterial and archaeal communities, we compared the ordinations obtained with both datasets using Procrustes analysis. There was a significant congruence between both datasets (Procrustes correlation, *R* = 0.48, *P* = 0.05; Figure 1C), most likely driven by the specificity of the prokaryotic community for each sponge type (HMA vs. LMA).

### Fungal community composition

**Figure 2:**
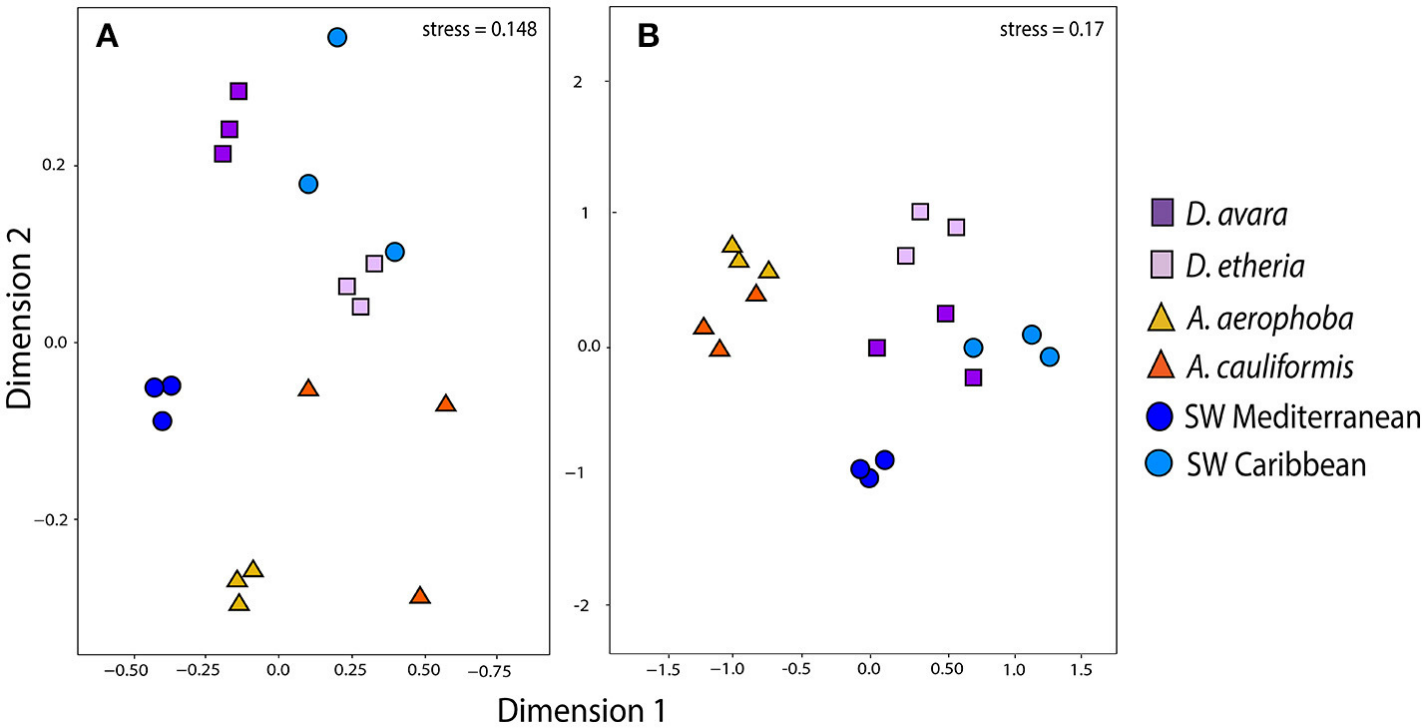
Community structure in eukaryotic communities. Ordination showing the first two axes of the NMDS analysis for **(A)** fungal OTU composition; **(B)** microeukaryotic OTU composition. Colors for each sample group are specified in the color legend.

After DNA sequence quality filtering and ITS2 extraction, a total of 574,040 sequences (816 OTUs, Supplementary Tables 11, 12) was found to be fungal and none of the sequences was detected as chimeric. Fungal community structure based on Bray–Curtis distances at the OTU level showed clustering patterns that were distinct for HMA and LMA sponge species (Figure 2A). The first axis of variation depicts a separation of the two geographic locations under study. The second axis shows a clear separation along sponge genus. The *A. cauliformis* samples were more spread out than those from its Mediterranean counterpart, but also separated on the second axis to the other Caribbean samples. The *D. etheria* samples appeared in close proximity to each other, but mixed with the Caribbean seawater samples: adonis analyses confirmed a significant difference in composition between sponge genera (adonis: *F* = 37.505, *R*^2^ = 0.654, *P* = 0.01) and along geography (adonis: *F* = 10.326, *R*^2^ = 0.090, *P* = 0.01). Fungal community structures revealed clustering by host sponge species and then by sponge genus (Supplementary Figure 1C), the effect being significant (PERMANOVA, *F* = 37.505; *P* = 0.01). On a third level of clustering, seawater samples from both geographic locations shared several OTUs with *Dysidea*, while the *Aplysina* samples formed a completely separate cluster.

**Figure 3:**
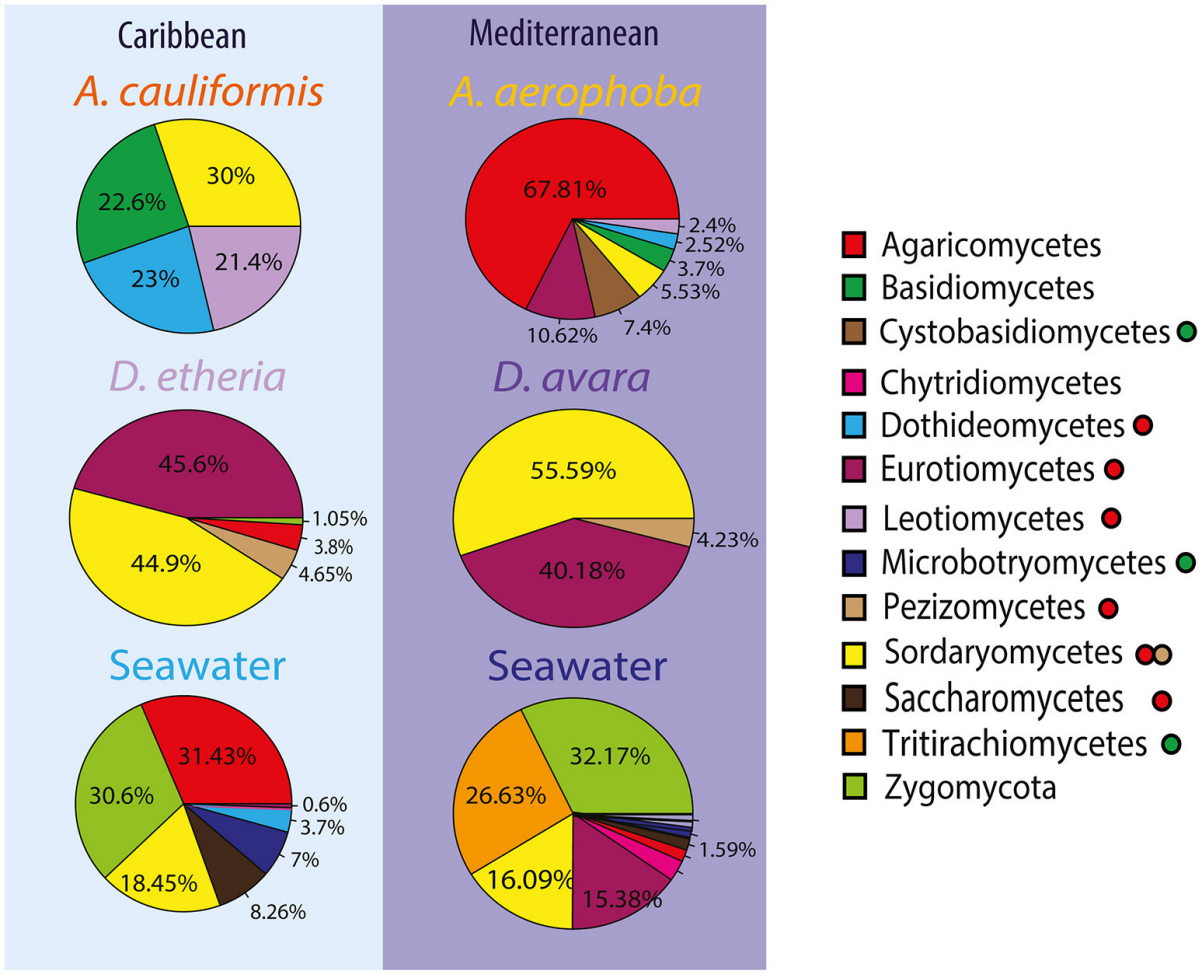
Compositional patterns of fungal communities using ITS2. Taxonomic overview of most abundant fungal divisions and classes across sample groups. The taxonomic overview shows class taxonomy where available. Colors for each fungal taxon specified in the color legend (squares). The fungal division for each class is further indicated with a filled circle after the name. Green was used to depict Basidiomycota without taxonomic assignments at a deeper taxonomic level; in a similar way, red was used for Ascomycota, and light brown was used for the subdivision Pezizomycotina.

Taxonomic assignments for fungal OTUs revealed substantial novelty. For instance, almost 60% of the sequences could not be identified further than Kingdom, that is, 418 OTUs were not assigned to any fungal division (Supplementary Tables 11, 12). Moreover, only 30% of the sequences were classified at family level, and 27% at genus level. Only OTUs enriched in a particular environment were further analyzed. OTUs that fell in the division Ascomycota dominated all sponge samples (Figure 3). The classes Sordariomycetes, Eurotiomycetes and Leotiomycetes were found most abundantly in the *Dysidea* samples regardless of geographic location (Figure 3). In *Aplysina*, up to 70% of OTUs were also assigned to Ascomycota, however the classes differed between the two species. *A. cauliformis* contained Sordariomycetes, Dothideomycetes and Leotiomycetes, while in *A. aerophoba* unknown Ascomycota were dominant. It is noteworthy that Basidiomycota appeared more in the *Aplysina* than in the *Dysidea* samples and more predominantly in *A. cauliformis*. In the seawater, sequences were mostly assigned to Ascomycota and Zygomycota (Figure 3). There were no enriched fungal OTUs at the genus level shared between *Aplysina* species (Supplementary Table 13). There was only one enriched OTU shared by both *Dysidea* genera, an unidentified Basidiomycete (Supplementary Table 14).

### Communities of microbial eukaryotes

After DNA sequence quality filtering, 7,550,789 non-chimeric reads were retained. Approximately 37% (2,787,534) of all reads were derived from sponges. The taxonomic classification of the sponge reads in each sample confirmed their host identities. After their removal 4,763,255 high-quality non-sponge eukaryotic sequences remained (3,517 OTUs). Fungi contributed with 470,919 (6.2%) of the reads. After applying a relative abundance threshold of 0.1%, 1,101 OTUs were kept (Supplementary Table 15). Members of 94 eukaryotic phyla and approximate phylum-level groups (not formally recognized) were detected in sponges and seawater from both geographic locations (Supplementary Table 16). In the case of eukaryotes, 42 OTUs were unclassified at the Phylum level (Supplementary Table 16), four of which were among the top 20 most abundant OTUs across samples (Supplementary Figures 4, 5).

Eukaryotic community structure based on Bray–Curtis distances at the OTU level showed a separation of clusters between (Figure 2B) HMA and LMA sponges and (Figure 2B) seawater samples (Figure 2B). This separation is picked up by the first axis of variation. The second axis separates sample origins (sponge species and geographic locations). However, these clustering patterns were not significant, neither for composition between sponge genera (adonis: *F* = 2.210, *R*^2^ = 0.102, *P* = 0.17) nor along geography (adonis: *F* = 1.745, *R*^2^ = 0.080, *P* = 0.07). In order to assess the overall similarity of eukaryotic communities associated with the sponges and surrounding seawater in both geographic locations, we performed a hierarchical clustering at the OTU level based on Bray–Curtis distance (Supplementary Figure 1D). This analysis revealed that there was no clear distinction between eukaryotic communities in seawater and those from sponges in either geographic location. Similarly, there was no distinctive clustering of all the specimens per habitat. In addition, there was no indication of geography-driven clustering of sponge-associated eukaryotic communities (Supplementary Figure 5). Finally, Procrustes analysis showed a moderate congruence between the bacterial and the microeukaryotic sequences (Procrustes correlation, *R* = 0.430), but it was not significant (*P* = 0.136).

## Discussion

### Bacteria and archaea

Bacterial communities associated with sponges are often found to follow a species-specific pattern, that is, (1) different individuals of the same sponge species in the same environment harbor similar bacterial communities, and (2) different sponge species from the same environment possess distinct bacterial communities (Webster et al., 2010; Thomas et al., 2016). In our study, the two HMA and LMA sponge species showed low interspecific variation in their associated bacterial, archaeal and fungal communities, clustering first by host species and then by host genus. Following our experimental design, where the same species in each genus were not present in both the Mediterranean and the Caribbean Sea, the statistical tests of geographic location is confounded with the species effect. We tried to alleviate this issue by performing adonis analyses, reversing the addition of terms to the models such that geographic location was considered first. In all cases where host species had previously been shown to be significant, it outweighed the geography effect. Therefore, we are confident, (consistent) with previous reports—at least in the case of Bacteria- that the sponge species effect is a main driver of community composition. It is a matter of debate whether both LMA and HMA sponges are equally capable of retaining their associated microbiota (Vacelet and Donadey, 1977; Kamke et al., 2010; Erwin et al., 2011; Schmitt et al., 2012; Giles et al., 2013). In general, HMA sponges have large and diverse microbial communities that relate to previously identified sponge-derived sequences, while LMA sponges have a microbiota with higher similarity to the seawater microbiota. Here, at 97% sequence similarity, and after removal of OTUs at < 0.1% relative abundance, we report richnesses between 681 OTUs (in *D. avara*) and 1302 OTUs (in A. *aerophoba*) (Table 1), matching expected differences in richness between LMA and HMA species. We find that although the bacterial communities in the *Dysidea* host species are generally less diverse compared to those in the *Aplysina* species, they are very specific in the communities they harbor, distinctive from the seawater samples (Figures 1A,B; Supplementary Figure 1).

The sponge genus *Aplysina* encompasses at least 42 accepted species as currently cataloged by the World Register of Marine Species. We included two of these species in our study, namely *A. aerophoba* and *A. cauliformis*. These are found in different habitats (Mediterranean and Caribbean, respectively). Based on the analyses at the OTU level, our findings are in line with previous reports for 16S rRNA gene clone libraries in other *Aplysina* species, identifying Cyanobacteria, Chloroflexi, Proteobacteria (Alpha- and Gamma-), Actinobacteria, Bacteroidetes and Acidobacteria as the most abundant bacterial phyla (Hardoim et al., 2009; Schmitt et al., 2011; Thomas et al., 2016). The diversity of the microbiomes of LMA sponges in our study is dominated by Proteobacteria, namely Alphaproteobacteria of the Rhodobiaceae family and unclassified Alphaproteobacteria. This finding matches previous work that reported that the phylum Proteobacteria was dominant in other LMA sponges. Examples are *Polymastia* sp. (Kamke et al., 2010), *Hymeniacidon heliophila* (Erwin et al., 2011) and *Crambe crambe* (Croué et al., 2013; Öztürk et al., 2013). We found that all bacterial OTUs shared between the *Aplysina* species (44) fall within the SC and/or SCC clusters (Simister et al., 2012) and a further examination using BLAST supported the matches to sponge- and/or coral-associated bacteria (Supplementary Table 6). These OTUs most likely constitute symbionts whose ancestors engaged in associations with sponges and corals prior to the divergence of the sponge/coral lineages involved. These are good candidates for further work targeting a functional understanding of the interaction between these (likely) symbiotic microbes and their hosts.

Regarding archaea, we did not find the sponge-specific archaeon “*Cenarchaeum symbiosum*” (Thaumarchaeota) (Hallam et al., 2006) in any of our sponge samples. The complete set of representative sequences of Archaea were checked via BLAST and no hit matched the accession number AF083072 which corresponds to the 16S rRNA gene for *C. symbiosum* (data not shown). Within the set of enriched OTUs in all sponge samples, we found a remarkable diversity for the Thaumarchaeota of the family Cenarchaeaceae (609 OTUs), and from its genus *Nitrosopumilus* (267 OTUs) (Supplementary Table 8, Supplementary Figure 3). Different OTUs within the Cenarchaeaceae appeared to drive the separation of the samples, following a host sponge-specific pattern. More OTUs unassigned at the genus level were common in *Aplysina* species, while *Nitrosopumilus* OTUs and further OTUs in the phylum Euryarchaeota were enriched in *Dysidea*, particularly in *D. etheria*. It is likely that the sponge habitat provides a suitable niche for species within the Cenarchaeceae to diversify. Earlier, two other Mediterranean sponges have shown enrichments for *Nitrosopumilus*, namely *Petrosia ficiformis* and *Corticium candelabrum* (Sipkema et al., 2015). Some species in this group are known ammonia oxidizers (Könneke et al., 2005), and it has been suggested that sponge-associated archaea may be involved in ammonia oxidation (Hoffmann et al., 2009) through the enzyme ammonia monooxygenase (amoA), probably performing a detoxification role in the sponge, which excretes ammonia as a waste product (Bell, 2008). Our work emphasizes novel archaeal diversity in sponges, in line with previous indications of uncharacterized archaeal diversity in sponges where taxonomic affiliations of *amoA* archaeal genes were poor, with only some clones affiliated to known ammonia oxidizers such as *Nitrosopumilus maritimus* and *Cenarchaeum symbiosum*, but mostly novel sequences within the Cenarchaeceae (Turque et al., 2010).

### Eukaryotic microorganisms

As compared to bacteria and archaea, the patterns and processes of fungal community composition in marine sponges are less well documented. Studies of wood-inhabiting marine fungi have shown that geographic location is the main factor determining fungal community composition (Rämä et al., 2014). In this study, we found a compositional difference in the fungal communities between sponge and seawater samples (Figure 3); however, the structure of the fungal communities is mainly driven by the sponge host and sponge genus (Figure 2). The potential for specific interactions between the sponges and their associated fungal communities has been described before. For instance, the vertical transmission of a unicellular fungus via the oocytes in the Mediterranean sponge *Chondrilla nucula* was described based on transmission electron microscopy (TEM) analysis (Maldonado et al., 2005). However, specificity of fungal communities in sponges had not been observed so far following the sponge-specificity concept developed for bacterial communities (Hentschel et al., 2002). Most likely due to the considerable depth of sequencing for fungal communities in this study (Table 1), we have been able to observe novel fungal diversity (Supplementary Table 12) and show that this diversity is largely determined by the host species and genus.

Using sequencing of a different marker, the V7-V8 region of the 18S rRNA gene, Naim et al. (2017) found similar fungal groups to be associated with sponges from the North Sea and the Mediterranean Sea, including *A. aerophoba*. Ascomycota clearly dominated the recovered community assigned to the division level. The 18S rRNA gene primers used in this study (Euk1Af and Euk516r) were chosen based on their former performance in broadly targeting the diversity of marine picoeukaryotes (Dìez et al., 2001; Wilms et al., 2006). Hence, the amplicons provide descriptive measures of both the fungal community and, more broadly, the eukaryotic community associated with sponges. The majority of 18S rRNA gene sequences was derived from the sponge hosts (on average, 85% of reads). In total, 821,333 eukaryotic reads were not assigned to Porifera in the sponges investigated. Even though we use only 15% of the generated reads, this number exceeds by far that of previous studies in which next generation sequencing technology was used, with respect to microeukaryote diversity in sponges (Simister et al., 2012; Rodríguez-Marconi et al., 2015). However, there is a limitation driven by the large data loss for the sponge samples (as reported in Table 1). The variability in the identified OTUs was large among samples, and within sponges of each species, most OTUs were only present in one or two out of the three replicates per group. Due to the low sample size, it becomes challenging to confidently discern specificity for the groups of microbial eukaryotes based on the 18S rRNA marker gene. Besides an increased sample size for future studies, we suggest designing primers that will preferentially amplify eukaryotes other than Porifera, thus preventing uneven data loss across sampled individuals.

A few previous studies also analyzed the 18S rRNA gene sequences obtained from eukaryotic microorganisms living within sponges. These studies reported protists (Alveolata, Mesomycetozoea, Rhizaria), protozoans (Amoebozoa, Apusozoa and Euglenozoa), protophytes (Cryptophyta, Rhodophyta, Viridiplantae, Cnidaria, Annelida and Stramenopiles (Webster et al., 2004; Sipkema and Blanch, 2009; Simister et al., 2012; Li et al., 2014) to be present. However, diatoms, dinoflagellates and fungi fulfilling the criteria for sponge-specific clusters (Simister et al., 2012) were not found in this study. Communities of eukaryotic microorganisms, with the exception of fungi, apparently did not assemble randomly, although no clear host- or genus-specific patterns could be discerned from the current study.

## Conclusion

The three-domain microbial communities from the LMA and HMA sponges of the genera *Aplysina* and *Dysidea* studied here are different from the surrounding planktonic communities, expanding previous observations for Bacteria. The correspondence between the low abundance of microorganisms and low phylum-level diversity in the LMA genus *Dysidea* was only true for bacterial communities, not so for microbes in the domains Archaea and Eukarya. We defined sets of bacterial OTUs that are enriched in particular sponge species and shared in each sponge genus, the latter indicating probable microorganisms that establish a symbiosis with each genus before the split of the sponge species studied here. These putative symbionts within each sponge genus are good candidates for future efforts to understand the functional roles that mediate their interactions with the sponge hosts. Furthermore, we present new diversity in the three domains of life. Especially in Bacteria and Fungi, these undescribed taxonomic levels are good candidates for bioprospection of associated microorganisms.

## Author contributions

MC, DS, JO and JDvE initiated and coordinated the project. MC, SH and BB planned and executed the sequencing and analysis pipelines for the project; MC, SH, and BB analyzed its outcome. MC wrote the paper, and all authors contributed to its improvement.

### Conflict of interest statement

The authors declare that the research was conducted in the absence of any commercial or financial relationships that could be construed as a potential conflict of interest.

## Supplementary material

Supplementary Figure 1 Community structure for samples assessed for all four microbial communities. Bray-Curtis dissimilarity of samples collected from the Mediterranean and the Caribbean Sea. OTUs with relative abundance > 0.1% were included in the analysis. **(A)** Bacteria, **(B)** Archaea, **(C)** Fungi, **(D)** microbial Eukarya.

Supplementary Figure 2 Bacterial phylum level relative abundance in the studied habitats for the top 19 most abundant phyla. Phyla as specified in color legend.

Supplementary Figure 3 Boxplots depicting Archaea OTUs found as significantly enriched in a given habitat. Log–relative normalized abundance per OTU. Colors show different habitats as specified in color legend. Padj: Adjusted *p*-value corrected for multiple comparisons with FDR.

Supplementary Figure 4 Relative abundance for the top 10 abundant phylum-level groups of eukaryotes found in this study, split based on sponge species habitat. Color legens as specified in figure.

Supplementary Figure 5 Heatmap for the top 100 most abundant microbial Eukaryotic OTUs. Bray-Curtis dissimilarity OTUs with relative abundance >0.1% were included in the analysis.

Supplementary Table 1 Metadata associated with samples used in this study.

Supplementary Table 2 Bacterial relative abundance table including OTUs above 0.1% in at least one sample.

Supplementary Table 3 Bacterial correspondences between OTU identifiers and assigned taxonomy using RDP.

Supplementary Table 4 Bacterial OTUs enriched in *Aplysina* species.

Supplementary Table 5 Bacterial OTUs enriched in *Dysidea* species.

Supplementary Table 6 Bacterial OTUs enriched in *Aplysina* and *Dysidea* host genera identified as belonging to sponge-specific clusters (SC) and/or sponge/coral-specific clusters (SCC).

Supplementary Table 7 Archaeal relative abundance table including OTUs above 0.1% in at least one sample.

Supplementary Table 8 Archaeal correspondences between OTU identifiers and assigned taxonomy using RDP.

Supplementary Table 9 Archaeal OTUs enriched in *Aplysina* species.

Supplementary Table 10 Archaeal OTUs enriched in *Dysidea* species.

Supplementary Table 11 Fungal relative abundance table including OTUs above 0.1% in at least one sample using ITS2.

Supplementary Table 12 Fungal correspondences between OTU identifiers and assigned taxonomy using the UNITE database.

Supplementary Table 13 Fungal OTUs enriched in *Aplysina* species.

Supplementary Table 14 Fungal OTUs enriched in *Dysidea* species.

Supplementary Table 15 Microbial eukaryotes relative abundance table including OTUs above 0.1% in at least one sample using 18S rRNA.

Supplementary Table 16 Microbial eukaryotes correspondences between OTU identifiers and assigned taxonomy using the SILVA database.
