# Supplementary figures and images for: Host Specificity for Bacterial, Archaeal and Fungal Communities Determined for High- and Low-Microbial Abundance Sponge Species in Two Genera
Source: Front Microbiol. 2017 Dec 20;8:2560. doi: 10.3389/fmicb.2017.02560 (PMC5742488; doi:10.3389/fmicb.2017.02560)

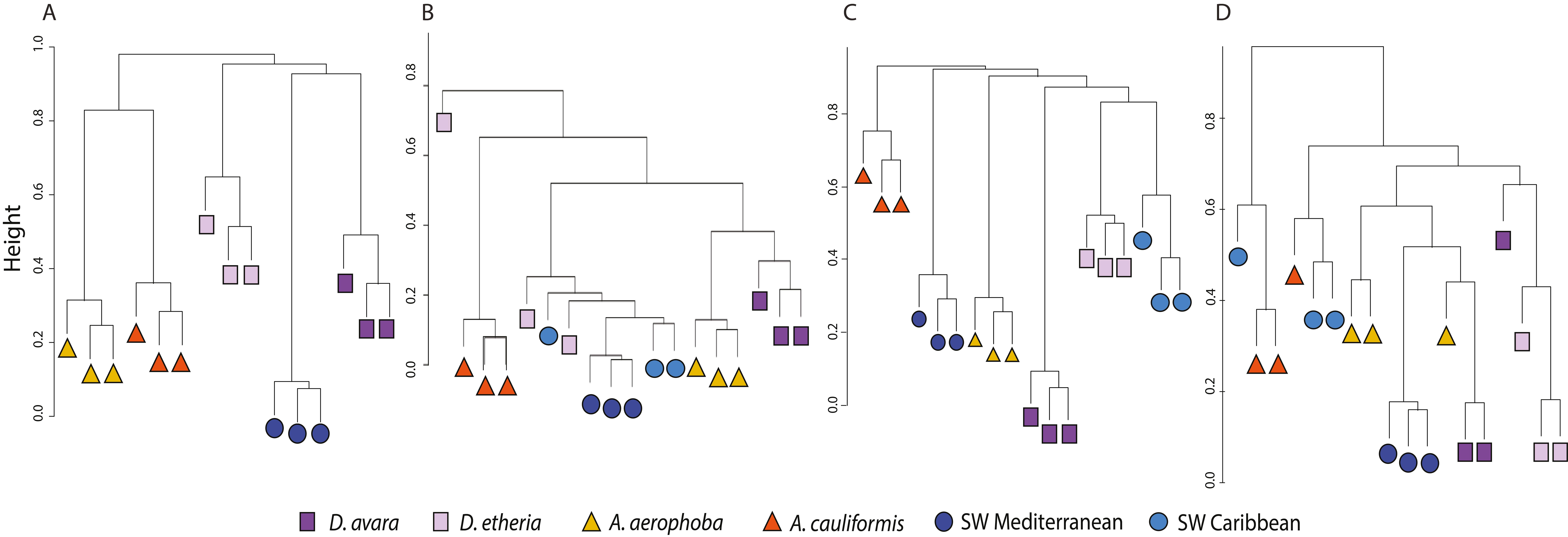

Supplement: Supplementary Figure 1 — Community structure for samples assessed for all four microbial communities. Bray-Curtis dissimilarity of samples collected from the Mediterranean and the Caribbean Sea. OTUs with relative abundance > 0.1% were included in the analysis. (A) Bacteria, (B) Archaea, (C) Fungi, (D) microbial Eukarya. [file Image1.JPEG]

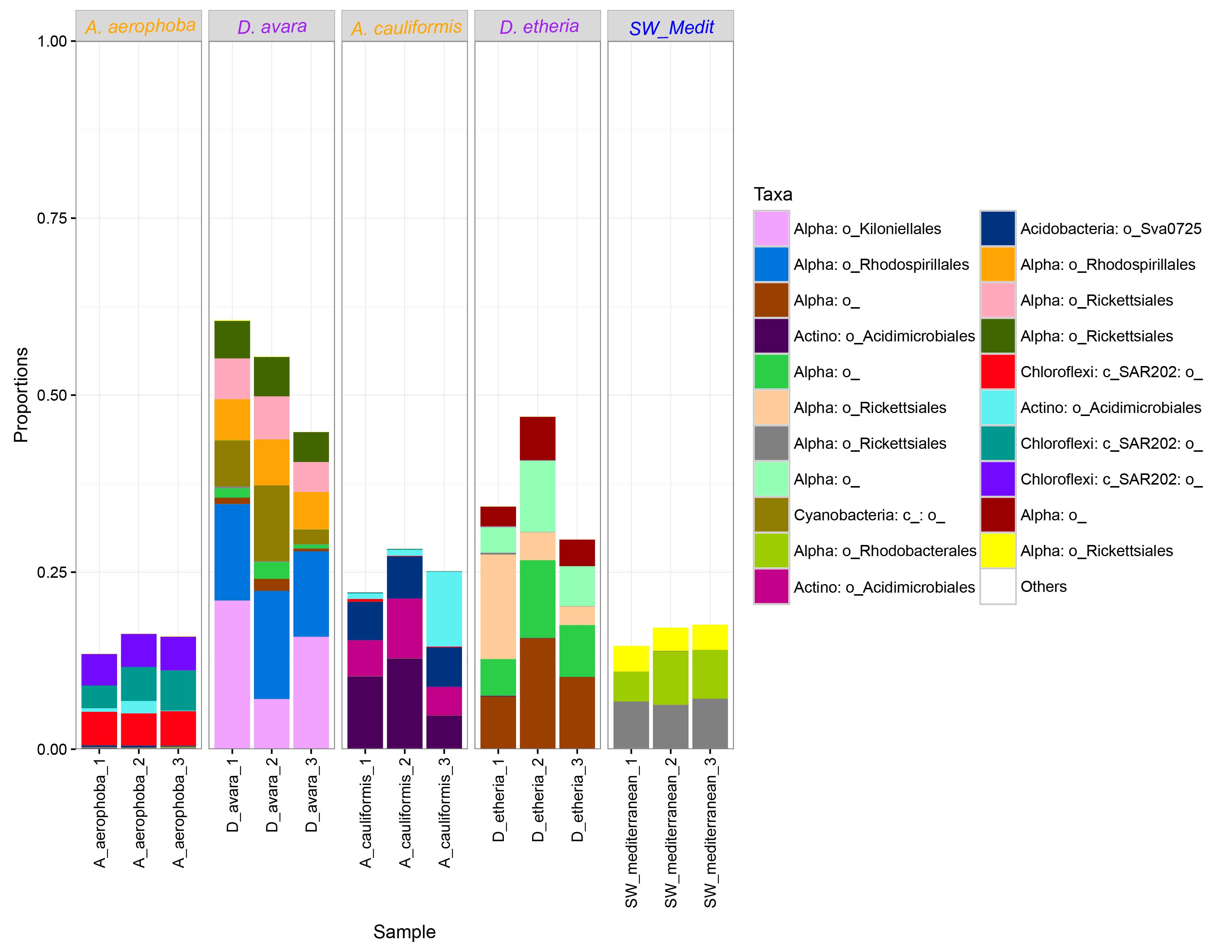

Supplement: Supplementary Figure 2 — Bacterial phylum level relative abundance in the studied habitats for the top 19 most abundant phyla. Phyla as specified in color legend. [file Image2.JPEG]

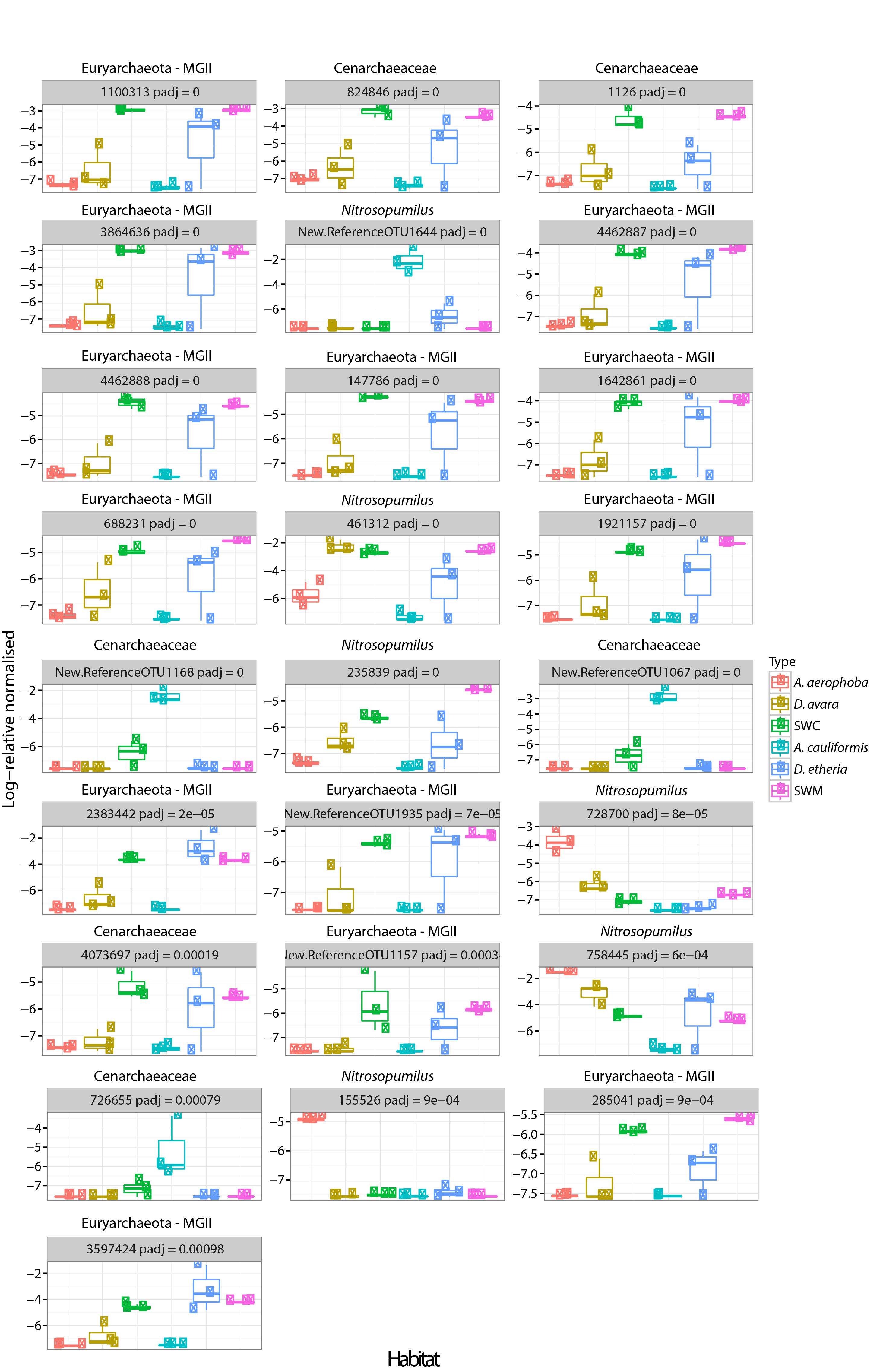

Supplement: Supplementary Figure 3 — Boxplots depicting Archaea OTUs found as significantly enriched in a given habitat. Log–relative normalized abundance per OTU. Colors show different habitats as specified in color legend. Padj: Adjusted p-value corrected for multiple comparisons with FDR. [file Image3.JPEG]

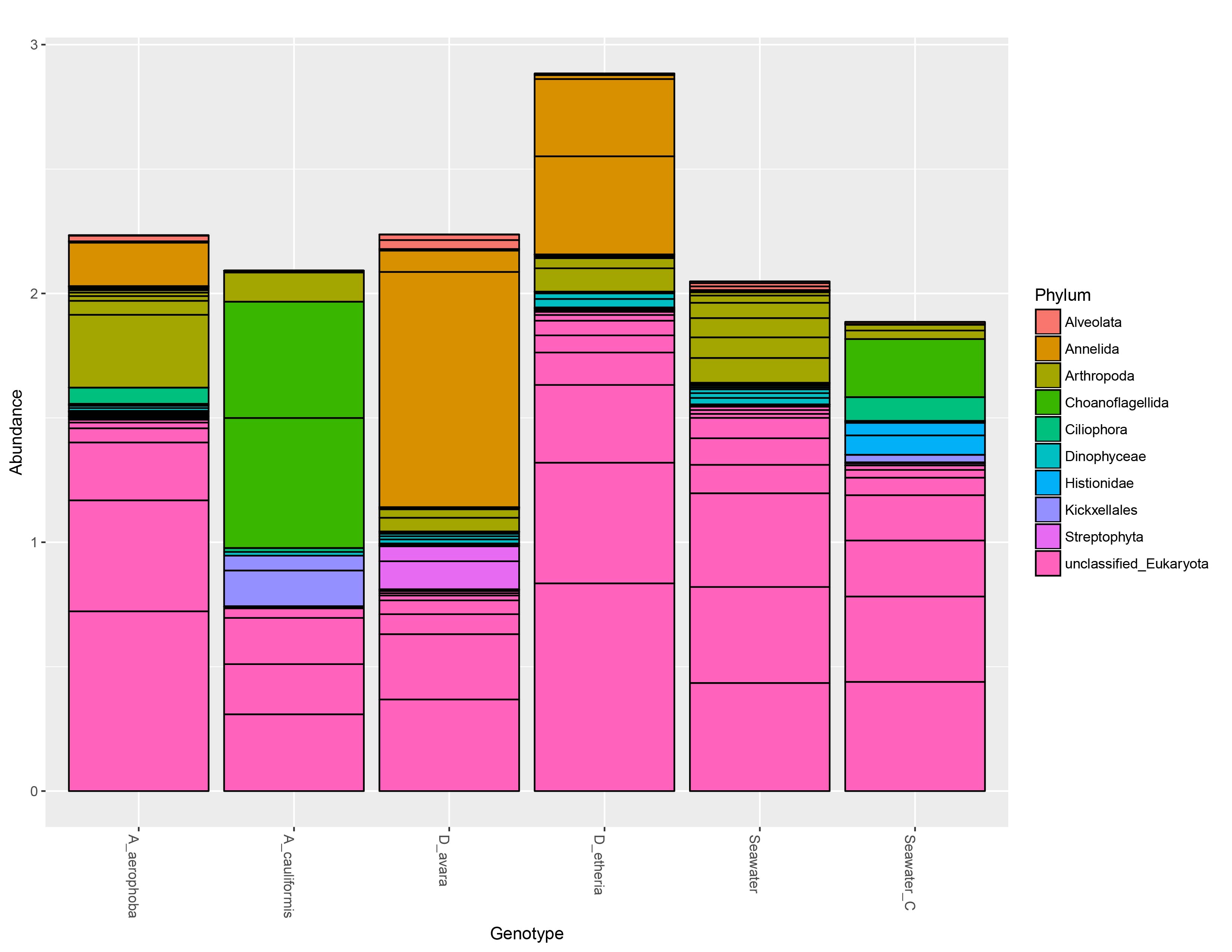

Supplement: Supplementary Figure 4 — Relative abundance for the top 10 abundant phylum-level groups of eukaryotes found in this study, split based on sponge species habitat. Color legens as specified in figure. [file Image4.JPEG]

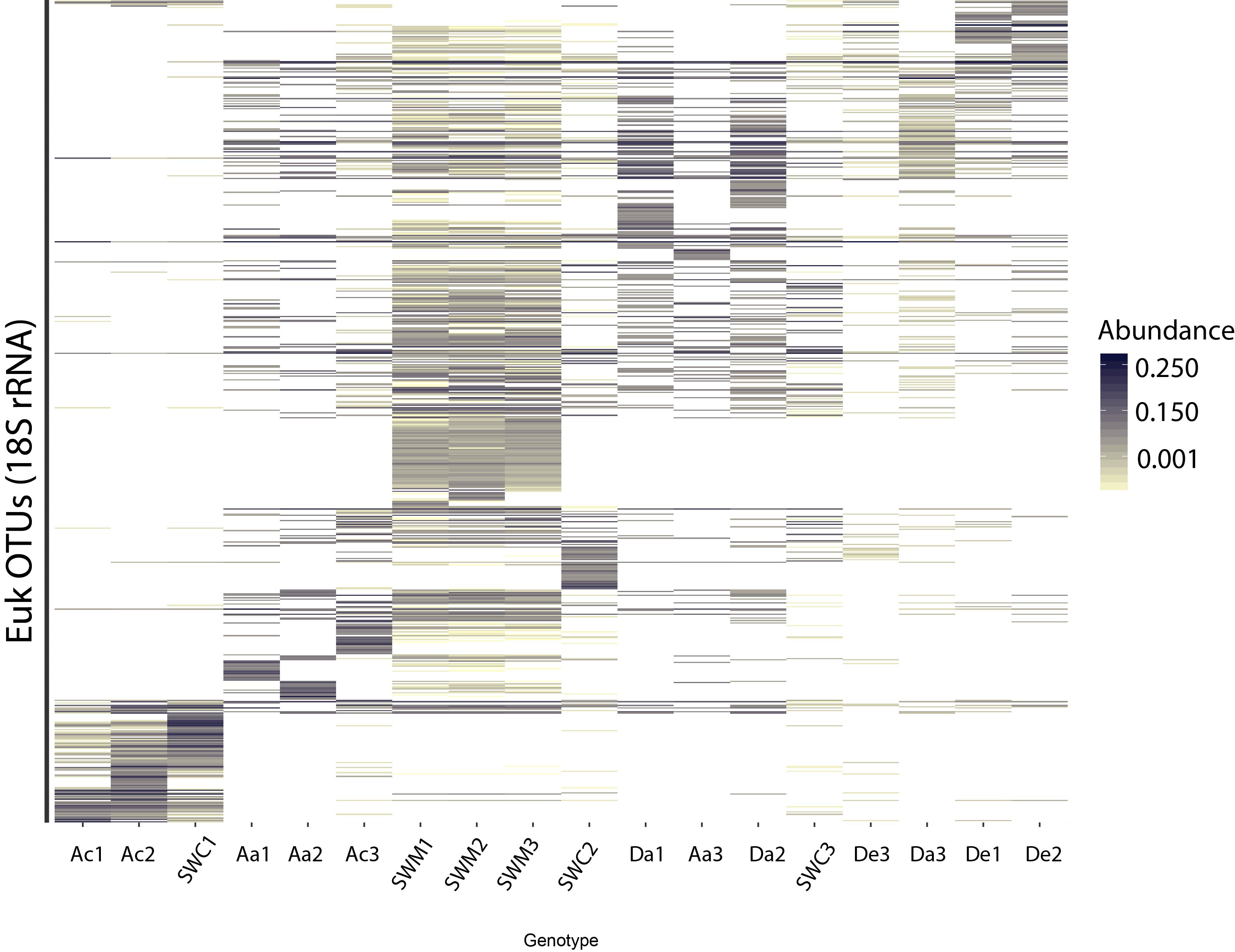

Supplement: Supplementary Figure 5 — Heatmap for the top 100 most abundant microbial Eukaryotic OTUs. Bray-Curtis dissimilarity OTUs with relative abundance >0.1% were included in the analysis. [file Image5.JPEG]
